# Supplementary material for: Identification of Transcriptional Networks Involved in De Novo Organ Formation in Tomato Hypocotyl Explants
Source: Int J Mol Sci. 2022 Dec 17;23(24):16112. doi: 10.3390/ijms232416112 (PMC9788163; doi:10.3390/ijms232416112)
Supplement: Supplementary file 1 [file ijms-23-16112-s001.zip › Supplementary_Larriba_TF.pdf]

Figure S1

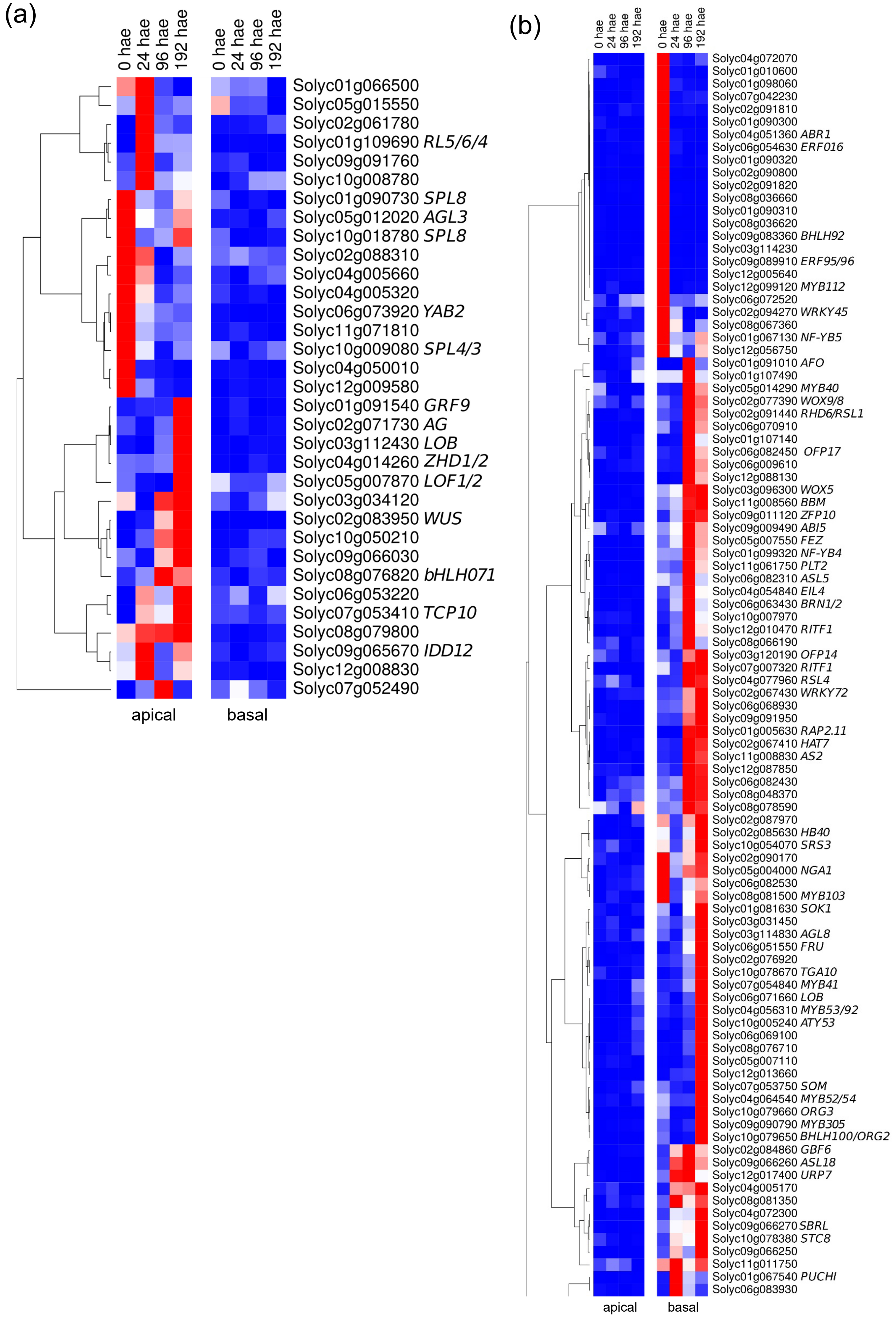

Figure S1. Hierarchical clustering of specific TF genes identified in the apical region (**a**) or the basal region (**b**) during *de novo* organ formation in tomato hypocotyl explants after wounding. Blue/white/red reflect normalized cpm values in each row, with red indicating the highest transcript abundance.

Figure S2

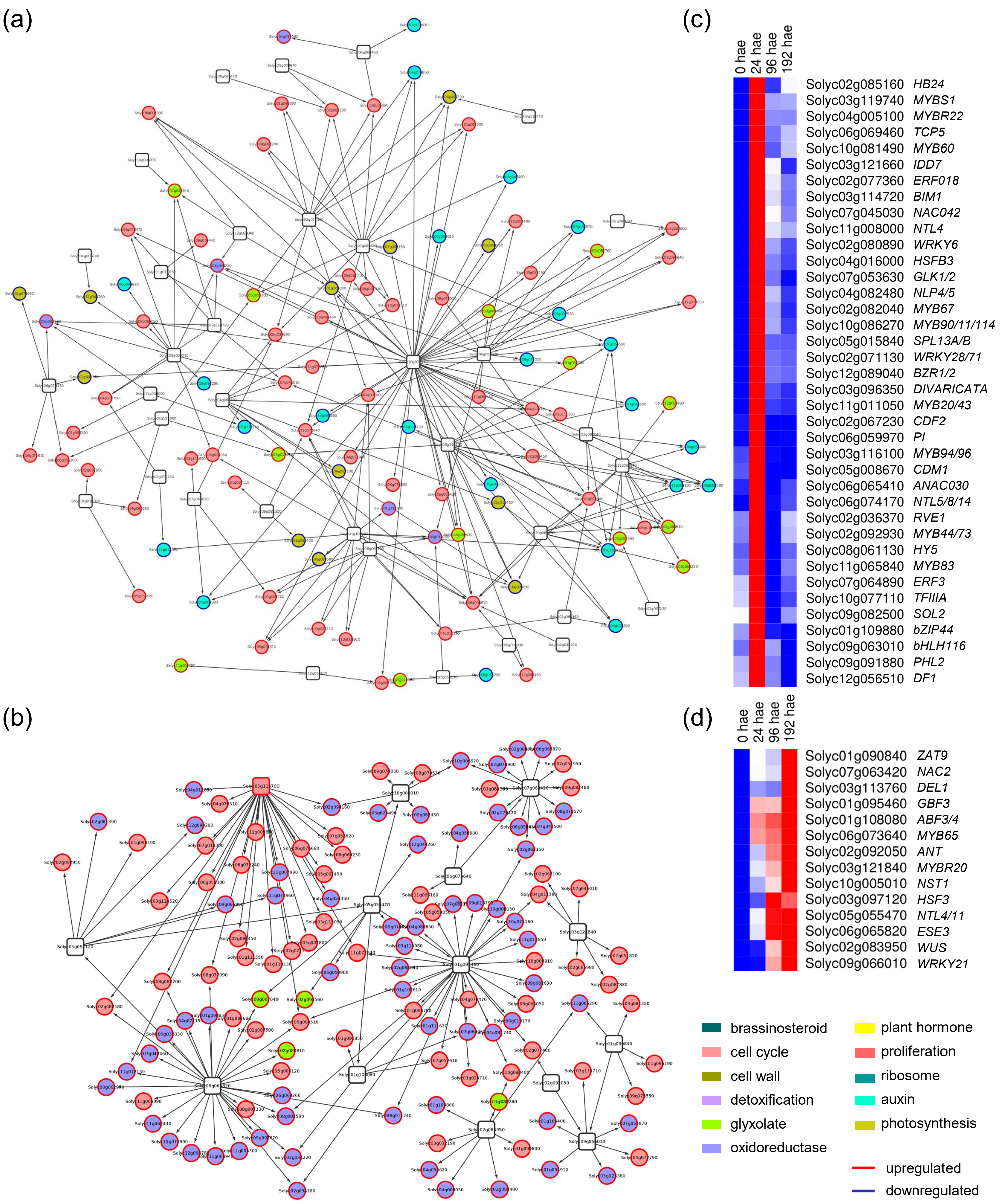

Figure S2. (**a–b**) GRNs for putative TF genes and their deregulated targets associated with P11 (a) and P13 (b) profiles in the apical region of the hypocotyl. (**c–d**) Hierarchical clustering over time of selected TF genes from P11 (c) and P13 (d) profiles. Blue/white/red reflect normalized cpm values in each row, with red indicating the highest transcript abundance.

Figure S3

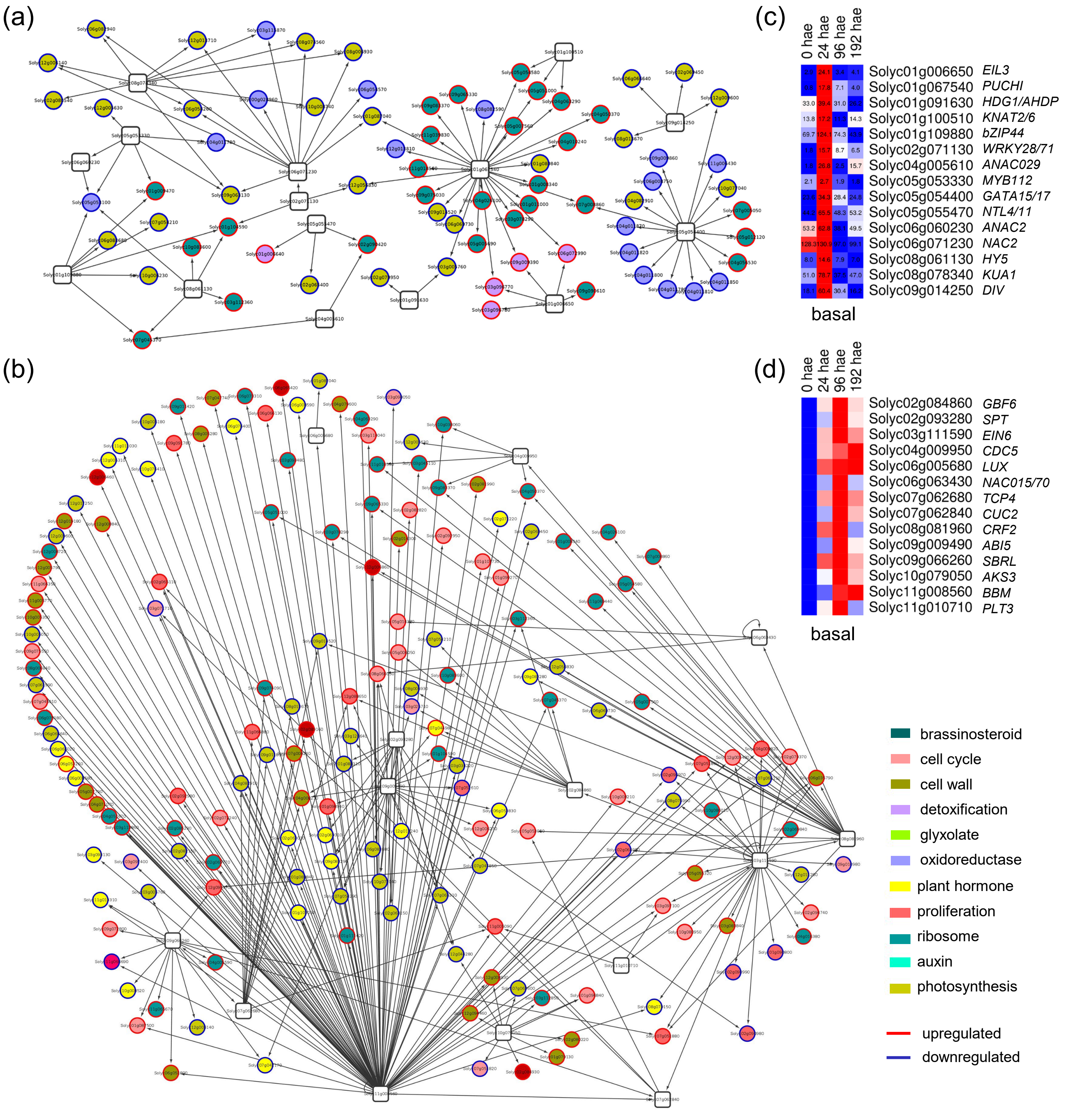

Figure S3. (**a–b**) GRNs for putative TF genes and their deregulated targets associated with P11 (a) and P15 (b) profiles in the basal region of the hypocotyl. (**c–d**) Hierarchical clustering over time of selected TF genes from P11 (c) and P13 (d) profiles. Blue/white/red reflect normalized cpm values in each row, with red indicating the highest transcript abundance.

Figure S4

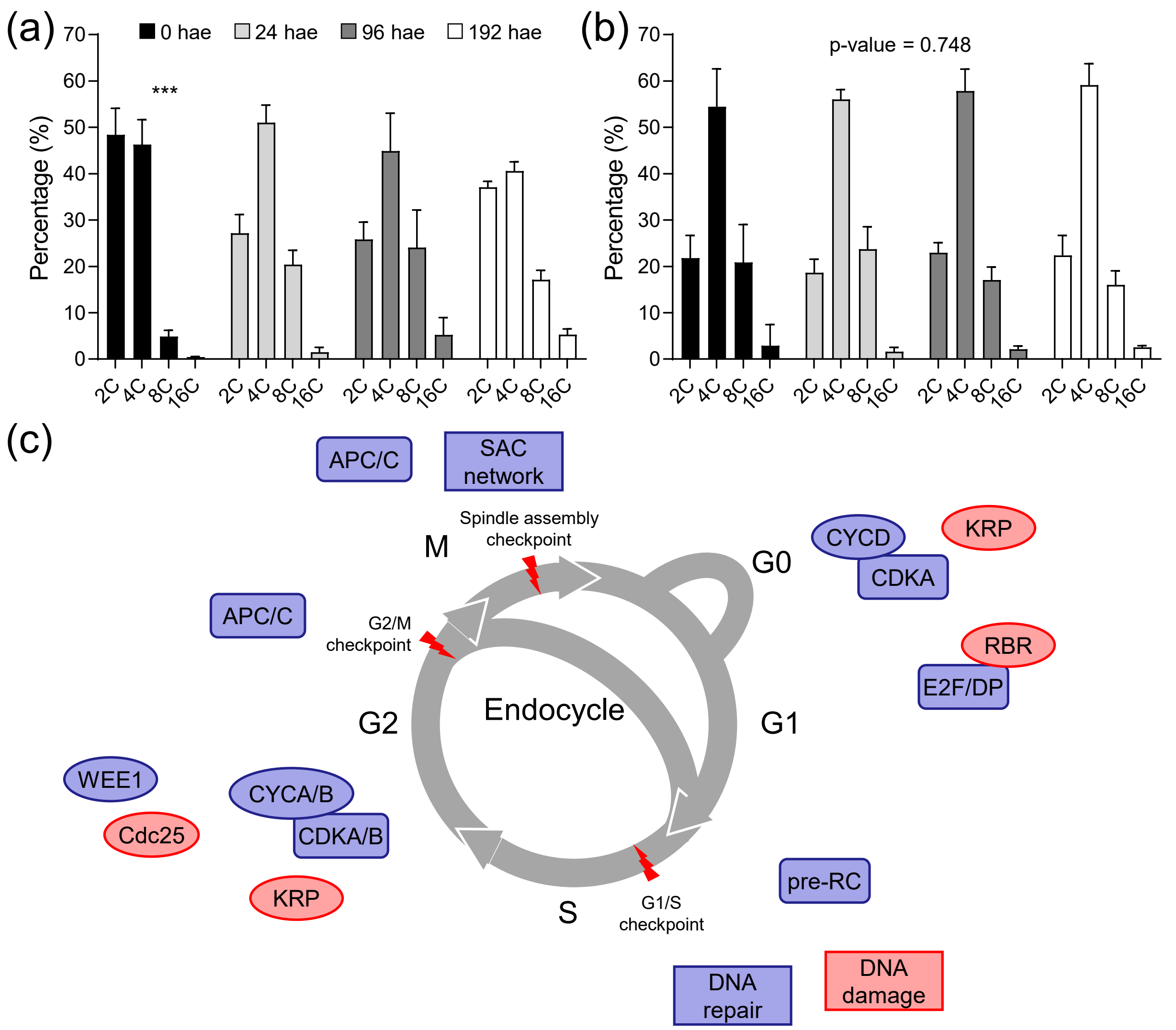

Figure S4. Cell cycle regulation during *de novo* organ formation (cont.). (**a**) Proportion of nuclei with different DNA content in the apical region of the hypocotyl explant during the studied time course. (**b**) Proportion of nuclei with different DNA content in the basal region of the hypocotyl explants during the studied time course. Asterisks in a–b indicate significant differences (p-value < 0.001, otherwise indicated) between samples. (**c**) Schematic diagram of the plant cell cycle, showing the core cell cycle genes families and pathways studied in this work (see Table S5).

Figure S5

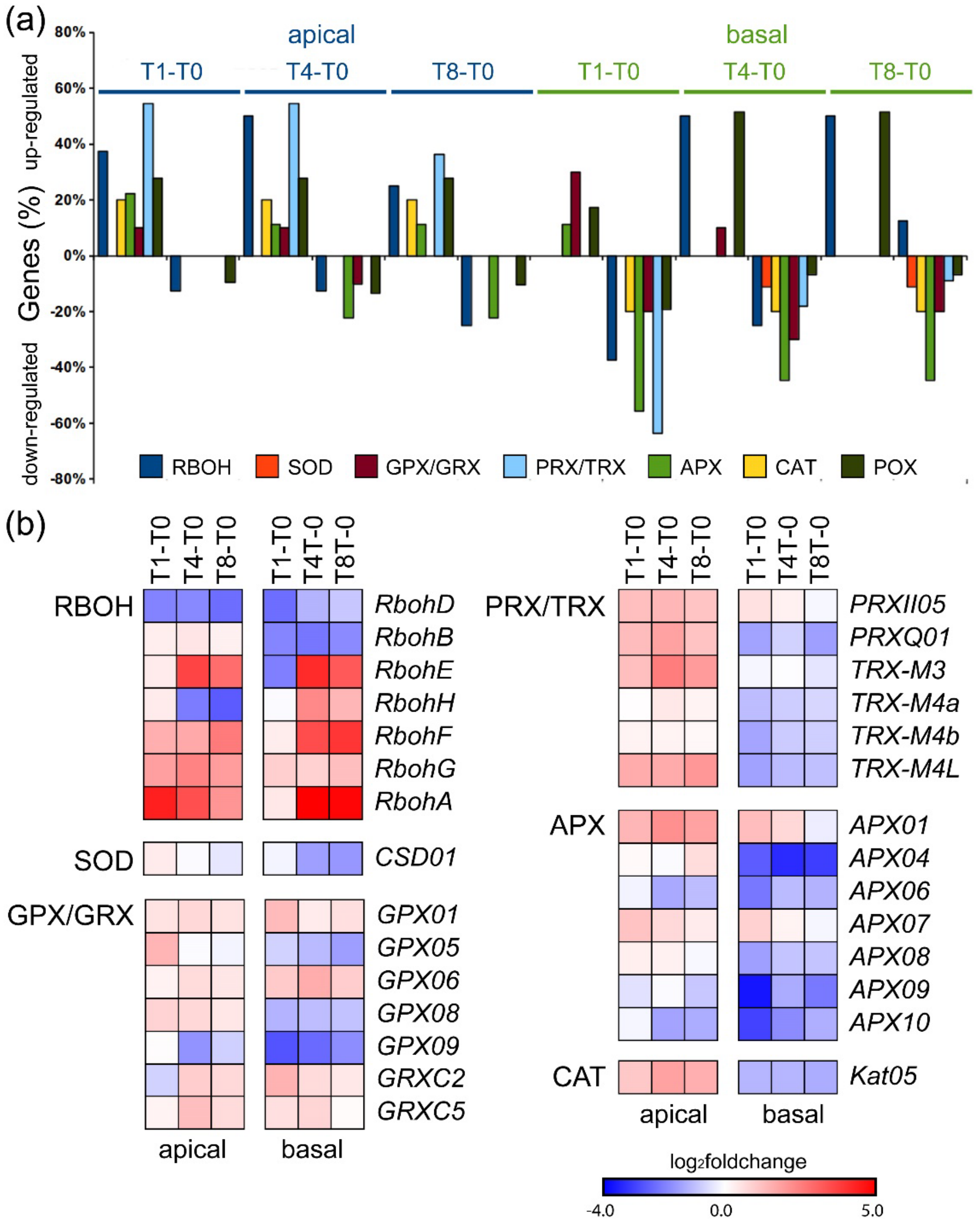

Figure S5. Expression of ROS related genes during wound-induced organ formation. (**a**) Percentage of deregulated genes of the studied oxidoreductases families in the apical and basal region of the hypocotyl. (**b**) DEG encoding ROS production and scavenging enzyme. RBOH, NADPH oxidase/respiratory burst oxidase homolog; SOD, superoxide dismutase; POX, peroxidase; GPX, glutathione peroxidase; PRX/TRX, peroxiredoxins/thioredoxins; APX, ascorbate peroxidases; CAT, catalases. Gene annotations are found in Table S6.

Figure S6

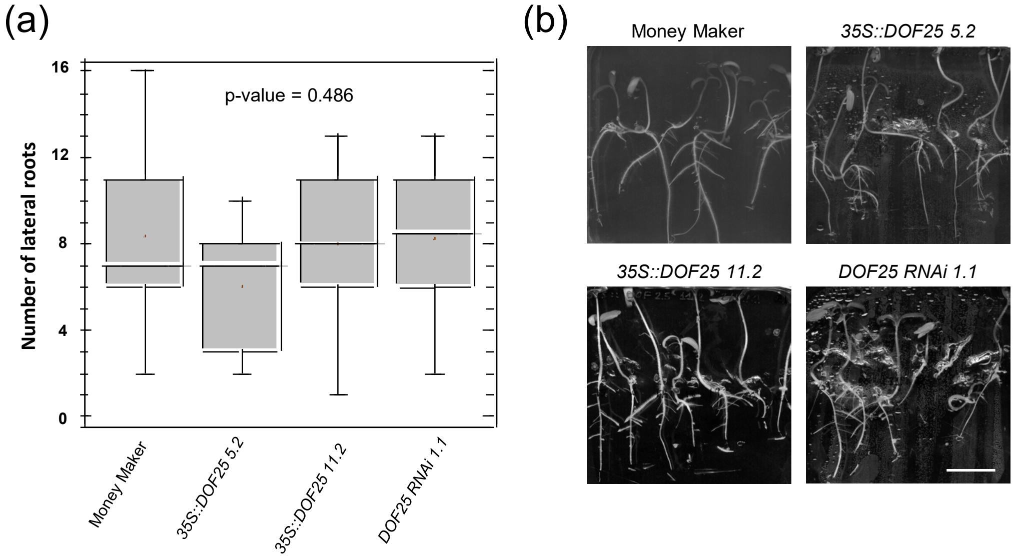

Figure S6. Wound-induced lateral root (LR) formation in *CDF3* tomato mutants. (**a**) LR number of seedlings after 3 days of apical root excision. (**b**) Representative images of seedlings after 3 days of apical root excision. Scale bars: 25 mm.

Table S1. Annotation and expression values of putative TF genes identified in tomato during *de novo* organ formation.

Table S2. Putative tomato TF genes whose *Arabidopsis thaliana* orthologs have been linked to tissue regeneration in previous studies.

Table S3. GO enrichment analyses of deregulated targets of TF genes found in selected gene expression profiles.

Table S4. Putative target genes of TFs from selected STEM expression profiles. In bold, the target genes common to both profiles of the same region (apical or basal) are indicated.

Table S5. Annotation and classification of expressed genes associated with core cell cycle regulation.

Table S6. Annotation and classification of genes associated with ROS production and detoxification. Gene annotation was retrieved from the indicated sources.
